# The impacts of partnering with cancer patients in palliative care research: a systematic review and meta-synthesis

**DOI:** 10.1177/26323524221131581

**Published:** 2022-10-18

**Authors:** Alessandra Paolucci, Ingrid Nielssen, Karen L. Tang, Aynharan Sinnarajah, Jessica E. Simon, Maria J. Santana

**Affiliations:** Department of Community Health Sciences, Cumming School of Medicine, University of Calgary, 3330 Hospital Dr NW, Calgary, AB T2N 4N1, Canada; Department of Community Health Sciences, Cumming School of Medicine, University of Calgary, Calgary, AB, Canada; Department of Community Health Sciences, Cumming School of Medicine, University of Calgary, Calgary, AB, Canada; Department of Medicine, Cumming School of Medicine, University of Calgary, Calgary, AB, Canada; Department of Community Health Sciences, Cumming School of Medicine, University of Calgary, Calgary, AB, Canada; Department of Family Medicine, Cumming School of Medicine, University of Calgary, Calgary, AB, Canada; Department of Medicine, School of Medicine, Queen’s University, Kingston, ON, Canada; Department of Oncology, Faculty of Medicine, University of Calgary, Calgary, AB, Canada; Department of Community Health Sciences, Cumming School of Medicine, University of Calgary, Calgary, AB, Canada; Department of Medicine, Cumming School of Medicine, University of Calgary, Calgary, AB, Canada; Department of Oncology, Faculty of Medicine, University of Calgary, Calgary, AB, Canada; Department of Community Health Sciences, Cumming School of Medicine, University of Calgary, Calgary, AB, Canada; Department of Pediatrics, Cumming School of Medicine, University of Calgary, Calgary, AB, Canada

**Keywords:** cancer, engagement, impact, palliative care, partnering, patient-oriented research, patient partner, systematic review, thematic meta-synthesis

## Abstract

**Background:**

Palliative care (PC) is an added layer of support provided concurrently with cancer care and serves to improve wellbeing and sustain quality of life. Understanding what is meaningful and a priority to patients, their families, and caregivers with lived experience of cancer and PC is critical in supporting their needs and improving their care provision. However, the impacts of engaging cancer patients *within* the context of PC research remain unknown.

**Objective:**

To examine the impacts of engaging individuals with lived experience of cancer and PC as partners in PC research.

**Methods:**

An a priori systematic review protocol was registered with PROSPERO (CRD42021286744). Four databases (APA PsycINFO, CINAHL, EMBASE, and MEDLINE) were searched and only published, peer-reviewed primary English studies aligned with the following criteria were included: (1) patients, their families, and/or caregivers with lived experience of cancer and PC; (2) engaged as *partners* in PC research; and (3) reported the impacts of engaging cancer PC patient partners in PC research. We appraised the quality of eligible studies using the Critical Appraisal Skills Program (CASP) and GRIPP2 reporting checklists.

**Results:**

Three studies that included patient partners with lived experience of cancer and PC engaged at all or several of the research stages were identified. Our thematic meta-synthesis revealed impacts (benefits and opportunities) on *patient partners* (emotional, psychological, cognitive, and social), the *research system* (practical and ethical) and *health care system* (service improvements, bureaucratic attitudes, and inaction). Our findings highlight the paucity of evidence investigating the impacts of engaging patients, their families and caregivers with lived experience of cancer and PC, as partners in PC research.

**Conclusions:**

The results of this review and meta-synthesis can inform the more effective design of cancer patient partnerships in PC research and the development of feasible and effective strategies given the cancer and PC context patient partners are coming from.

## Introduction

Both challenges and opportunities within health care services can be strategically leveraged to advance knowledge and strengthen the quality and provision of patient care. Including and prioritizing patients’ voices, needs, and urgencies, and partnering with them in health research that informs practice and care is instrumental to achieving holistic person-centered care. The evidence is now substantive and wide-ranging that the inclusion of patient and community members within the health research continuum can inform health care policy and practice, and benefit patients, researchers, research ecologies, and the health care system.^1,2^ Moreover, patient and community-informed research yields more inclusive research results that can be accessed and implemented sooner, and in more universal, useable, and equitable ways.^1,2^

Language about the inclusion of ‘patients’ and ‘community members’ *collaborating as partners on health-*related research teams is variably defined and described globally and across participatory action research (PAR) methodologies.^2–5^ Current international initiatives are presented in Table 1 to highlight these nuances. We will adopt the Canadian Institutes of Health Research’s (CIHR) Strategy for Patient-Oriented Research (SPOR) framework of patient engagement (PE) – the active and meaningful collaboration of patients as *partners* in any and/or all phases of the research continuum^1^ – to promote clarity and consistency surrounding the concept of engaging patients as partners.

**Table 1.** Participatory action research (PAR) initiatives and approaches.

Initiative	Year	Country	PAR approach	Definition	Partner term	Partner role	Stages of research cycle	Ethics approval required
National Institute for Health and Care Research (NIHR) INVOLVE	1996	United Kingdom	Patient and public involvement (PPI)	Research that is conducted ‘“with” or “by” members of the public rather than “to”, “about”, or “for” them’^18^	Public, service users	Consultation, collaboration, and/or user controlled research	Identifying and prioritizing; commissioning; designing and managing; undertaking; disseminating; implementing; evaluating impact	No
National Health and Medical Research Council (NHMRC)	2002	Australia	Consumer and community engagement	Active, non-tokenistic partnerships throughout the research journey that benefits all and can lead to quality research that meets the needs of the community and promotes the translation of research into improved policy and practice^19^	Consumer, community members	Involvement should be in a minimum of four key phases (i.e. **determining** research priorities; **development** of research and study design; **research**, including participant recruitment, ethics and governance, and oversight or governance of conduct of research; **reporting**, communications and publication, including translation, implementation strategies/activities or identification of future research.^19^	Building relationships; developing the research idea; developing the project and seeking agreement; collecting data; analyzing the data and making sense of the findings; report writing; sharing and translating the results into action; learning from experience^19^	No
International Collaboration for Participatory	2009	Germany	Participatory health research	‘The goal is to maximize the participation of those whose life or work is the subject of the research in all stages of the research process . . . research is not done “on” people as passive subjects providing “data” but “with” them to provide relevant information for improving their lives. The entire research process is viewed as a partnership between stakeholders which may include academic researchers; professionals in the fields of health care, education and social welfare; members of civil society; policy makers and others’^20^	People, stakeholders, civil members of society	‘The stakeholders decide which questions will be asked in the research, what the goals of the research are, how the research will be done and how the results will be used’^20^		No
Health Research Board (HRB)	2010	Ireland	Public, patient, and carer involvement in research	HRB uses NIHR’s definition of PPI. ‘By “involvement” we mean the active involvement between people who use services, carers, the general public and researchers’^18,21^	Public, patient, carer	It does not include the use of people as participants in research (or as research ‘subjects’) and does not provide data for individual research projects^21^		No
Patient-Centered Outcomes Research Institute (PCORI)	2010	United States of America	Patient and stakeholder engagement in research	‘The meaningful involvement of patients, caregivers, clinicians, and other health care stakeholders throughout the entire research process – from planning the study, to conducting the study, and disseminating results’^22^	Patients, patient partners, caregivers, clinicians, and other health care stakeholders	‘Patient partners provide – the lived experience as a person with an illness or injury or the caregiver or family member of such a person – is incredibly valuable, and contributions of these partners should be recognized accordingly . . . Patients and other health care stakeholders are equitable partners – as opposed to research subjects – who leverage their lived experience and expertise to influence research to be more patient centered, relevant, and useful’^22^	‘Engagement often occurs along a continuum ranging from stakeholder input, to consultation, to collaboration or shared leadership’^22^	No
Canadian Institutes of Health Research (CIHR) Strategy for Patient-Oriented Research (SPOR)	2015	Canada	Patient-oriented research (POR)	Defines POR as a continuous process of engaging patients in multi-disciplinary research focused on identifying patient priorities to improve health outcomes and health care system practices. In POR, patient engagement (PE) includes the active and meaningful collaboration of patients as *partners* in any and/or all phases of the research continuum^1^	Patient	The role of patients as partners on POR teams involves offering their lived experience and expertise to inform and advise on research priorities and processes^1^	Priority setting and planning; development of the research proposal; scientific review; ethics review; oversight of a research project; recruitment of research participants; data collection; data analysis and interpretation; knowledge exchange and translation; evaluation and quality assurance.^1^	No
Community-Based Participatory Research (CBPR); Israel *et al.*^23^	2001		Community-based participatory research (CBPR)	CBPR is defined as a collaborative approach distinguished by the active engagement of all partners and in all aspects of the project. CBPR is often used to include marginalized or often excluded populations in grass-roots research about issues and challenges that affect them. CBPR requires patients to serve as both partners and participants throughout *all* stages of the research^23^	Co-researchers	Community members join health research teams as ‘co-researchers’ and work together with research teams both as partners and participants. Inform research priorities and processes, as well as contribute data.^23^	Including priority setting, data collection, data analysis, and dissemination of results^23^	Yes

Despite the lack of consensus in language across the international landscape, engaging patients as partners moves patients beyond the traditional view of mere data contributors (i.e. participants) to a more involved collaboration; the research focus shifts from being ‘on or about’ those impacted to research ‘with’ *those the research impacts. Existing primary studies and synthesis reviews have reported on the impacts of partnering with patients*, *with various disease-specific lived experiences (e.g. acute*, *chronic*, *cancerous*, *non-*cancerous)^6–9^ and across various health care contexts (e.g. non-palliative,^10,11^ palliative).^12^ However, to our knowledge, there are no studies that have studied the impacts across multiple contexts (i.e. cancer *and* palliative).

Palliative care (PC) is an added layer of support provided concurrently with cancer care and serves to improve wellbeing and sustain quality of life.^13^ A new public health approach to PC, involving PAR, has been recommended to address the needs of people with complex and advanced conditions.^14^ While previous studies report on how cancer patients, or PC patients, separately, have served as partners, the impacts of engaging patients with lived experience of both cancer *and* PC *within* the context of PC research remains unknown. Current knowledge gaps exist regarding whether patients with lived experience of cancer and PC have unique, disease-specific needs and experiences affecting their involvement in serving as research partners compared with patients with other health conditions (e.g. asthma, diabetes, cancer). Understanding what is meaningful and a priority to patients, their families, and caregivers with lived experience of cancer and PC is critical in supporting their needs and improving their care provision.

The objective of this systematic review is to examine the impacts of engaging patients with lived experience of cancer and PC as partners in PC research. We aim to learn how individuals with lived experience of cancer and PC have been engaged as partners in PC health research projects and to understand the impacts of this engagement on: (1) patient partners, (2) research projects, and (3) health care systems. We will use the findings from this knowledge synthesis to generate recommendations for the future design and conduct of meaningful research within cancer *and* PC research.

## Methods

This systematic review’s protocol has been registered with PROSPERO (CRD42021286744) and the review has been reported according to the Preferred Reporting Items for Systematic Reviews and Meta-Analyses (PRISMA) 2020 statement.^15^

### Adopted definitions and framework

CIHR’s SPOR^1^ definitions of a patient, PE, and patient-oriented research (POR) were adopted for this review. A ‘patient’ refers to an individual (e.g. patient, family member, friend, caregiver) with lived experience of cancer *and* PC.^1^ Thus, we refer to patient partners with lived experience of cancer and PC as Cancer Palliative Care Patient Partners (CPCPP). Our definition of ‘patient’ is linked and comparable with other studies using PE in Alberta.^7,16^ We used and modified the Art and Humanities Research Council’s (AHRC)^17^ definition of ‘impacts’ to include anything that influenced the research and affected patient partners and/or researchers at an individual, community, and/or policy development level.

We used the SPIDER framework^24^ to operationalize our research question. The sample included adult patients, and/or their families and caregivers with lived experience of cancer and PC. Our phenomenon of interest was the involvement of this sample as *partners* in PC research. We anticipated that through our review we would obtain studies that were qualitative or mixed-methods in design and evaluate the *impacts* of involving CPCPPs in the PC research process.

### Applied methods of PE

Based on the scope and aim of our systematic review, we determined that it would not be appropriate to partner with patients in any stage of our study.

### Search strategy

A systematic search strategy was developed with the support of a health research librarian in MEDLINE and translated to three other electronic databases (i.e. APA PsycINFO, CINAHL, and EMBASE) on November 14, 2021 (Table 2). Four comprehensive concepts related to our question of interest were combined to obtain relevant search results. Each query was comprised terms related to one of the following concepts: (1) PC, (2) patient/family/caregiver, (3) patient-oriented and PE research, (4) impacts, and (5) cancer. Our search strategy included two search combinations, one with and without the cancer concept. We imported results based on the search strategy without cancer and manually screened for the concept of cancer to ensure a more comprehensive search. We identified additional candidate studies from the reference lists of eligible studies and excluded gray literature and conference abstracts. Only English language full-text primary studies, regardless of publication date, were considered for inclusion.

**Table 2.** OVID MEDLINE search strategy.

Database	Search strategy	Total
OVID	1. Palliative care	58,865
MEDLINE	2. terminal care or hospice care	35,998
	3. (palliative care or hospice care or palliative treatment* or terminal care or palliative medicine or hospice nursing or palliative nursing or palliative supportive care or end-of-life or end of life or end of life care or end-of-life care).tw,kf.	64,535
	4. 1 or 2 or 3	109,651
	5. Patients or inpatients or outpatients/	63,913
	6. (patient* or client* or inpatient* or outpatient*).tw,kf.	7,489,744
	7. Family	80,883
	8. (family or famil* or family member* or relative*).tw,kf.	2,619,816
	9. Caregivers	43,468
	10. (caregiver* or care giver* or family caregiver* or spouse caregiver* or carer*).tw,kf.	93,443
	11. 5 or 6 or 7 or 8 or 9 or 10	9,462,286
	12. (impact* or outcome* or evaluat* or benefi* or harm* or challeng*).tw,kf.	7,545,172
	13. (patient-oriented research* or PPE or PPI or co-research* or co-build* or co-creat* or co-design*).tw,kf.	31,406
	14. (research* adj5 (participat* or involv* or engag* or partner*)).tw,kf.	50,241
	15. Research/	203,023
	16. 13 or 14 or 15	280,774
	17. (cancer* or oncolog* or neoplasm* or carcinoma*).tw,kf.	2,648,763
	18. exp Neoplasms	3581,375
	19. 17 or 18	4,273,810
	20. 4 and 11 and 12 and 16 and 19	178
	21. animals/not humans/	4,890,312
	22. 20 not 21	178
	23. 4 and 11 and 12 and 16	473
	24. 23 not 21	473

### Study eligibility and selection

Following the execution of the search strategy, search results from each electronic database were imported to Covidence.^25^ Two reviewers (AP and IN) independently screened titles and abstracts in duplicate, met regularly to discuss any eligibility criteria-related inquiries that arose, and consulted a third senior reviewer (MS) to resolve any disagreements. Full-text screening followed a similar procedure and full agreement was obtained.

Study eligibility criteria are highlighted in Table 3. We only excluded titles and abstracts, and full-text studies if: (1) only patients with lived experience of non-cancer illnesses and/or PC were engaged; (2) patients with lived experience of cancer and PC were solely engaged as *participants* instead of *partners* in PC research; (3) patients with lived experience of cancer and PC were engaged in research evaluating PC services; or (4) if they evaluated a PC *intervention*. Therefore, we only included published, peer-reviewed primary studies in the English language that aligned with our inclusion criteria: (1) adult patients, their families, and/or caregivers with lived experience of cancer and PC; (2) engaged as *partners* in PC research; and (3) reported on the impacts of engaging CPCPPs in PC research. We also included articles if there was a mix of patient partners with lived experience of cancer and non-cancer illnesses.

**Table 3.** Systematic review eligibility criteria.

Inclusion criteria	Exclusion criteria
1. Studies focused on *patients* (i.e. patients, their families, and/or caregivers) with lived experience of cancer and palliative care (PC)	1. Studies focused on *patients* with lived experience of non-cancer illnesses and/or PC only
2. Studies focused on *engaging* patients with lived experience of cancer and PC as partners in PC research	2. Studies focused on patients with lived experience of cancer and PC solely as *participants* rather than *partners*
3. Studies that applied patient-oriented and patient engagement research methods	3. Studies that engaged patients with lived experience of cancer and PC in *cancer care* research and/or PC services
4. Studies that reported findings on the impacts of engaging patients with lived experience of cancer and PC in PC research	4. Studies reporting on the evaluation of a PC *intervention*
5. English language studies only	5. Secondary data (i.e. commentaries, editorials, letters, no methods, no findings, no extractable data)

Patient *partners* were differentiated from *participants* if they assisted in carrying out the study at any point (e.g. research planning, development, recruitment, data collection, analysis, dissemination, knowledge translation) during the research process and did not contribute any data. Studies that did not fit these criteria were not included. In addition, there were no limits for the following: (1) the study publication date; (2) study design (i.e. qualitative, quantitative, mixed-methods); (3) geographical context; or (4) patient partner characteristics (e.g. sex, gender, age, type of cancer diagnosis, stage of patient’s cancer).

### Data extraction and quality assessment

Data were extracted from studies that met our inclusion criteria using a standardized form developed based on the protocol. One reviewer (AP) pilot tested this data extraction form with a single included study and then extracted data on the remaining included studies. Subsequently, a second reviewer (IN) verified the extracted data for accuracy and comprehensiveness. Data extracted from each study were related to three domains: (1) *study demographics* (i.e. year of publication, country study was conducted); (2) *study information* (i.e. research questions, aims/objectives; theoretical underpinnings; research type; study design; patient partners’ characteristics including sex, age, sample size, type of patient partner, type of cancer diagnosis, stage of patient’s cancer; study methodology and methods; data analyses; key study findings; reported study strengths, limitations, future research directions and recommendations stated by the authors); and (3) the *impacts* (e.g. on patient partners, researchers, the research, and/or health care system) *of cancer PE in PC research*.

To maintain consistency in our approach, the quality of all included studies was independently assessed by two reviewers (AP and IN) using two evidence-based assessment tools. Any discordance was resolved through discussion or by a third senior reviewer (MS). The qualitative methodological quality of included studies was assessed using the Critical Appraisal Skills Program (CASP).^26^ To assess the quality, transparency, and consistency of the international PE evidence base reported by included studies, the Guidance for Reporting Involvement of Patients and Public (GRIPP2) short-form checklist was used.^27^ Despite our anticipation of eligible mixed-methods studies, we did not encounter any studies using this design that fit our inclusion criteria and, therefore, did not need to use the Mixed-Methods Appraisal Tool (MMAT) Version 2018.^28^

### Data synthesis

Given all the studies that met our eligibility criteria were qualitative, the heterogeneity of, and lack of reported empirical data in our included studies, precluded the statistical pooling of findings for a meta-analysis and any subgroup analyses. Instead, study characteristics were tabulated and narratively synthesized to integrate and explore relationships within the data. We used a qualitative meta-synthesis approach;^29^ specifically, a thematic synthesis (i.e. line-by-line coding of primary study findings; the development of descriptive and analytical themes)^30^ to thematically translate, integrate, and describe all relevant qualitative findings from our eligible studies.^30^ One reviewer (AP) independently and inductively coded each line of verbatim text (e.g. original authors’ findings) that reported impacts of PE in cancer PC research. Themes were developed by systematically ‘going beyond the findings’ of eligible studies and generating additional concepts or understandings.^30^ Team discussions were held to check for interpretation consistency, further refine emergent descriptive and analytical themes, and verify themes in relation to our review aim to ensure robustness.

## Results

### Description of included studies

#### Study flow

**Figure 1.**
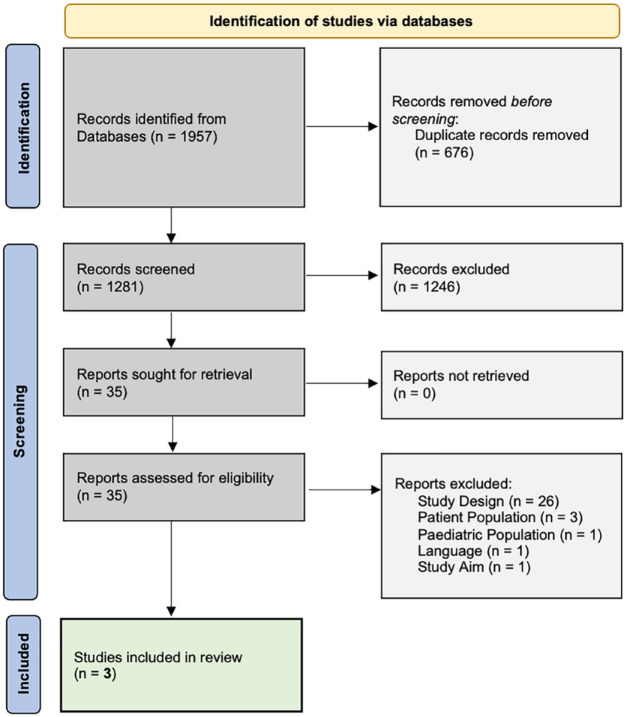
PRISMA (2020) flow diagram of the systematic review process.

Overall, a total of 1,957 possibly relevant studies were identified through the four electronic databases: APA PsycINFO (*n* = 212), CINAHL (*n* = 486), EMBASE (*n* = 791), and MEDLINE (*n* = 472). Covidence removed 676 duplicates leaving a new total of 1,281 potentially eligible studies for titles and abstract screening. Of these, only 35 full texts underwent retrieval, uploading, and secondary screening. The primary reasons for excluding most of the full-text studies included: partnering with a non-cancer and/or pediatric population; using PE language incorrectly (e.g. if patients were described as ‘partners’ or ‘co-researchers’ but did not actually collaborate on research processes and only contributed data that were subsequently analyzed by researchers without lived experience expertise); employing a non-PAR study design; or study aims that were not focused on PE impacts. A total of three studies were included in the final data extraction and synthesis phase. Figure 1 displays the PRISMA flow diagram of the selection of articles through the different phases of the systematic review.

#### Study characteristics

We identified three studies^31–33^ that engaged CPCPPs at any or all the research stages defined in patient and public involvement (PPI)^6^ and POR.^34^ As highlighted in Table 4, the included studies were published between 2006 and 2016, and all three studies: (1) gathered data in the United Kingdom; (2) implemented a PPI approach; (3) employed a qualitative study design; and (4) were situated within non-Indigenous cultural contexts.

**Table 4.** Study characteristics.

Authors	Country	Participatory action research (PAR) type	Study design	Study methods
Wright *et al.*^31^	United Kingdom	PPI	Qualitative	Focus groups and questionnaires
Cotterell *et al.*^32^	United Kingdom	PPI	Qualitative	Focus groups and survey questionnaire
Forbat *et al.*^33^	United Kingdom	PPI	Qualitative	Interviews

### Patient partner and engagement characteristics

#### Demographics

Of the three included studies, the number of patient partners recruited to engage in the PC research were 2,^32^ 4,^33^ and 15.^31^ While most studies reported on the sex of their participants, Forbat *et al.*^33^ study was the only one to report on both the sex and age of their patient partners (i.e., females, over 18 years of age). Thus, most studies did not report on patient partner characteristics, including race and ethnicity.

#### Lived experiences

As aligned with our inclusion criteria, all individuals involved as partners in each study had lived experience of both cancer *and* PC. Aside from Forbat *et al.*^33^ mentioning that their patient partners were 2 years post-bereaved, the authors of all three studies did not describe patient partners’ years of cancer and PC lived experience. Regarding the *type* of patient partners involved, Cotterell *et al.*^32^ did not specify, while Forbat *et al.*^33^ partnered with *former* carers, and Wright *et al.*^31^ partnered with both patients and carers but did not specify whether their carers were current, former, or both. Similarly, Wright *et al.*^31^ and Cotterell *et al.*^32^ did not report who their partners cared for, while Forbat *et al.* stated that their patient partners cared for ‘their parents (*n* = 3), spouse (*n* = 1), and child (*n* = 1)’^33^ (p. 762). Except for Wright *et al.*^31^ mentioning that two of their patient partners were in receipt of PC services, Cotterell *et al.*^32^ and Forbat *et al.*^33^ did not describe whether their partners were currently experiencing cancer and PC. In addition, none of the three studies provided information regarding the type of cancer(s) patient partners were affiliated with. Only Wright *et al.*’s^31^ study stated patient partners were affiliated with advanced-stage cancer.

#### Roles and stages of engagement

The authors of each included study referred to their patient partners as ‘co-researchers’^31,33^ and ‘service user researchers’.^32^ Table 5 displays the focus of each included study, the process for patient partner involvement, patient partner roles or contributions, and the levels of engagement and impact (i.e. on the patient partners, research projects, and health care system). Included studies reported a spectrum of PE in research activities (i.e. priority setting and planning, development of the research proposal, scientific review, ethics review, oversight of a research project, recruitment of research participants, data collection, data analysis and interpretation, knowledge exchange and translation, and evaluation and quality assurance), which we aligned with CIHR’s research lifecycle^34^ in Table 6. Wright *et al*.’s^31^ reference-group members were engaged with the study design and development of patient information sheets; later, they joined as patient and carer co-researchers. Cotterell *et al.*^32^ involved service user researchers and a research advisory, comprised experienced researchers in service user involvement, and patients with lived experience of cancer and PC who advised on the study and data interpretation.

**Table 5.** Patient partner characteristics in included studies.^a^

Authors	Study Focus	Process for patient partner involvement	Patient partner role or contribution	Level of engagement^b^	Impact
Wright *et al.*^31^	(1) To discuss techniques used in study to identify, train, and involve people at the end of life as collaborators in research. (2) To describe the experiences of working with patients receiving palliative care services, reference will be made, where appropriate, to other patients collaborating in the study who are not receiving PC. (3) To focus on the challenges of involving patients in end-of-life research and makes recommendations on how they can be managed. (4) To explore cancer patients’ views and attitudes toward cancer research, and (5) to identify their priorities	Reference group advised on the design of the study and related materials. Reference-group members volunteered to become ‘co-researchers’ and received training and support to collaborate throughout the course of the study. Co-researchers co-moderated focus groups^31^	Study planning; data collection	Consult, involve	Patient partners, research, health care system
Cotterell *et al.*^32^	Aimed to describe the impacts of involvement on the lives of service users affected by cancer who participate in involvement activities	‘Research Advisory Group members and service user researchers provided advice and support throughout the study. Early themes were shared with the Research Advisory Group and their insights fed into the analytic process. Service user researchers co-developed the outline research bid and full application for funding and collaborated with writing the research documentation for participants; planning recruitment; developing the questions to ask; carrying out interviews and focus groups; analyzing data; and in dissemination activities, which included the writing of a booklet based on findings. Service user researchers individually read all transcripts and noted initial interpretations’^32^	Study planning and recruitment; co-development of study documents and materials; data collection and analysis; knowledge dissemination	Consult, Involve	Patient Partners, health care system
Forbat *et al.*^33^	To explore what data emerge when caregivers are trained to interview current caregivers about their experiences	Co-researchers were recruited and trained in conducting qualitative interviews. Co-researchers interviewed participants. Co-researchers were involved in refining the research question and interview schedule, participating in initial thematic analysis and aiding dissemination through conference presentations. Co-researchers co-developed the semi-structured interview guide and piloted the guide with the research team^33^	Study planning; data collection and analysis; knowledge dissemination	Involve	Patient partners, research, health care system

a Table column headings replicate those used by Bird *et al.*^35^

b Level of Engagement framework adopted from Manafo *et al.*^2^

**Table 6.** Summary of stages of patient engagement in palliative care research lifecycle.

First Authors	Priority setting and planning	Development of the research proposal	Scientific review	Ethics review	Oversight of a research project	Recruitment of research participants	Data collection	Data analysis and interpretation	Knowledge exchange and translation	Evaluation and quality assurance
Wright *et al.*^31^	X						X	X		
Cotterell *et al.*^32^	X	X				X	X	X	X	
Forbat *et al.*^33^	X						X	X	X	

### Research partnership characteristics

#### Recruitment strategies

The three studies employed different strategies for identifying and recruiting patient partners. Wright *et al.*^31^ stated that they developed a reference group through patient forums of UK cancer networks where members later joined as co-researchers. In addition, co-researchers were identified from a participating hospice day care service using a targeted approach and through collaboration with a hospice clinical team.^31^ Forbat *et al.*^33^ only reported opportunity sampling by recruiting their co-researchers from carer organizations, while Cotterell *et al.*^32^ did not specify how their patient partners were identified and recruited.

#### Training and compensation

As one of CIHR’s^1^ guiding pillars of PE, *support* of patient partners in research collaborations can be reflected in the training opportunities offered and provided to patient partners. Wright *et al.*^31^ reported individually and group training (i.e. 90 minutes long) their co-researchers. In addition, Wright *et al.*^31^ held mock focus groups with their co-researchers and provided additional sessions upon request to accommodate co-researchers while receiving PC services. Forbat *et al.*^33^ reported training their co-researchers in conducting qualitative interviews and trained interview respondents who chose to join as a co-researcher. Forbat *et al.*^33^ also mentioned that co-researchers were not instructed regarding the extent to which they should share details about themselves or ‘what not to say’. By contrast, Cotterell *et al.*^32^ did not report on any training of their service user researchers. Forbat *et al.*^33^ and Cotterell *et al.*^32^ did not mention providing their patient partners with any financial compensation, and while Wright *et al.*^31^ reported that financial resources were given to patient partners, they did not provide any details regarding what compensation was given.

### Quality reporting assessment

#### Qualitative methodology assessment

Quality appraisal scores for the qualitative methodology of all included studies are shown in Table 7. As all three included studies utilized a qualitative study design, Cotterell *et al.*^32^ and Wright *et al.*^31^ used focus groups, while Forbat *et al.*^33^ employed interviews. Two reviewers (AP and IN) independently assessed whether each of the studies appropriately reported each of the following sections: (1) a clear statement of aims, (2) suitable qualitative methodology, (3) research design, (4) recruitment strategy, (5) data collection, (6) researcher-participant relationship considerations, (7) ethical considerations, (8) rigorous data analysis, (9) clear statement of findings, and (10) extent of the study’s value. Studies by Wright *et al.*^31^ and Forbat *et al.*^33^ demonstrated high quality across all ten domains; Cotterell *et al.*^32^ demonstrated high quality across nine of ten domains but did not clearly report on any ethical considerations. Given the congruence between the qualitative components of the included studies, a *strong* overall quality reporting score resulted for each study.

**Table 7.** Quality appraisal of qualitative design in included studies using CASP (2018) Checklist.^26^

First Authors	Clear aims statement	Appropriate qualitative methodology	Appropriate research design	Appropriate recruitment strategy	Appropriate data collection methods	Researcher–participant relationship considerations	Ethical considerations	Rigorous data analysis	Clear statement of findings	Extent of research value
Wright *et al.*^31^	Y	Y	Y	Y	Y	Y	Y	Y	Y	Y
Cotterell *et al.*^32^	Y	Y	Y	Y	Y	Y	N	Y	Y	Y
Forbat *et al.*^33^	Y	Y	Y	Y	Y	Y	Y	Y	Y	Y

*Note*: Y, Yes; CT, Can’t Tell; N, No.

#### PE reporting assessment

A quality assessment of the reporting of PE in our included studies can be found in Table 8. Again, for each of the included studies, two reviewers (AP and IN) independently assessed five components of quality PE reporting: (1) aims, (2) methods, (3) study results, (4) discussions and conclusions, and (5) reflections and critical reflections of PE. Since the studies by Forbat *et al.*^33^ and Wright *et al.*^31^ were the only two studies to addressed each of these components, we allocated a *strong* quality score for them both. Although Cotterell *et al.*^32^ reported on four of the five components, they did not provide discussion, conclusions, or critical reflections on their PE. As a result, we gave this study a *moderate* quality score.

**Table 8.** Quality appraisal of patient and public involvement (PPI) reporting in included studies using GRIPP2 Short-Form Checklist (2017).^27^

First Authors	Aim	Methods	Study results	Discussion and conclusions	Reflections/critical perspective
Wright *et al.*^31^	821	821–822	822–825	825	825–826
Cotterell *et al.*^32^	161	162, 163	163–166	166–168	NR
Forbat *et al.*^33^	760	760–762	762–766	766–767	766–767

*Note*: Page numbers are displayed to indicate the page each component is reported on; NR, not reported.

### Thematic meta-synthesis on patient partnership impacts

**Figure 2.**
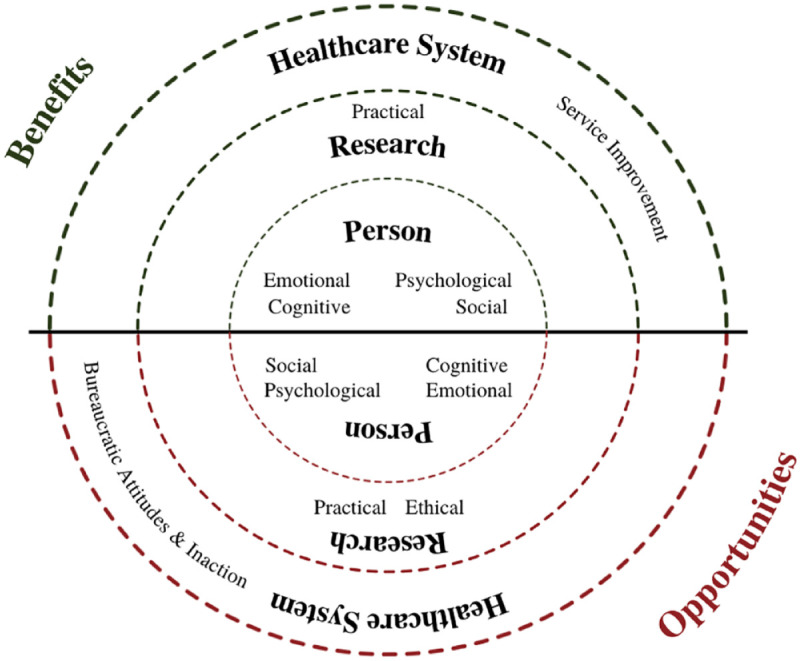
Emergent model from meta-synthesis.

Reported impacts related to cancer PC patient partnerships in PC research from each of the included studies were thematically synthesized into *benefits* (i.e. anything that was identified as a positive influence, produced a helpful outcome, or promoted the wellbeing of the CPCPPs, researchers, research, and/or the health care system) and *opportunities* (i.e. anything that was identified as a need for improvement or to advance the wellbeing of the CPCPPs, researchers, research, and/or the health care system). As illustrated in Figure 2, eight sub-themes emerged from our thematic meta-synthesis.

#### Impact on patient partners

Partnering with cancer PC patients in PC research has been associated with various *emotional*, *psychological*, *cognitive*, and *social* experiences. Impacts to each of these four domains will be discussed in relation to the benefits, opportunities, or both.

##### Emotional

Several benefits surrounding CPCPPs’ emotional health and wellbeing were synthesized across the included studies. Emotional benefits, as an emergent sub-theme, can include living with purpose, having a sense of personal achievement, being able to express emotions in a safe environment, and displaying emotional agility. Some CPCPPs viewed the collaborative approach in PC research as offering them an opportunity to make active and meaningful contributions – for their own benefit as they approached the end of their lives, and for those coming after them in utilizing PC services and the research community.^32^ PE in cancer PC research encouraged CPCPPs to live with purpose, ‘be part of shaping new and more appropriate treatment for others going through a similar experience’, and *pay back* service to the health professionals and system that treated and supported them.^32^ Taken together, CPCPPs described their engagement as helping them ‘live well with cancer’ and ‘refocus their lives in a positive, purposeful, and productive way’ by supporting their cancer survivorship.^32^

CPCPPs also shared a few engagement opportunities regarding their emotional wellbeing. Some felt they were unable to achieve all they desired in their engagement roles given a lack of clarity, vision, aim, and understanding about their involvement and role expectations.^32^ This invoked frustration and emotional distress among CPCPPs collaborating in PC research. Other CPCPPs reported emotional burdens related to ‘hearing accounts and discussing their own personal experiences’,^31^ compounded by cancer discussions with clinicians and researchers who came across as negative, insensitive, and dismissive of CPCPPs’ needs. Some health professionals seemed void of emotion when discussing project and health care-related issues and information that were profoundly emotional to CPCPPs.^32^ Thus, some research teams ensured emotional supports were available to CPCPPs.^31^

##### Psychological

Numerous psychological benefits were reported by the included studies. Psychological health and wellbeing can include sense of self, self-confidence, mental health, and inspiration and motivation. CPCPPs reported a sense of enhanced self-confidence based on their research involvement, sensing all that they could achieve, and sharing their lived experiences with others.^32^ Partnering in PC research also enabled CPCPPs to negate the effects of their cancer illness while their overall mental health improved. For instance, CPCPPs reported their involvement was ‘a positive way to keep active, combat depression and loneliness, and deal with their cancer diagnosis and treatment’, dispel feelings of hopelessness, and help them differentiate between the *disease* and the *person*.^32^

CPCPPs’ research involvement also positively impacted study participants. During interviews, participants viewed CPCPPs as role models for their perseverance and engagement in research.^32^ This inspired both participants and CPCPPs to ‘fight’ their cancer. CPCPPs were highly motivated to improve PC services and make meaningful contributions to PC research.^32^

A critical opportunity related to patient partners’ engagement and psychological wellbeing involved one’s concept of self in relation to one’s diagnosis. ‘It cannot be assumed that co-researchers are at ease in conducting research with other patients on account of their diagnosis alone’^31^ (p. 824). It may be demotivating and a trigger for CPCPPs to relive their ‘trauma’, especially with those who do not share in their lived experiences. Patient partners with lived experiences of both cancer and PC are recommended.^31^

##### Cognitive

Cognitive health and wellbeing are promoted through continuous learning and involve displaying attentiveness, information processing, and mental flexibility to carry out daily activities. Collaborating with CPCPPs in PC research created learning opportunities and a positive space for participants and CPCPPs to engage in enriching discussions.^32^ When CPCPPs co-interviewed participants, a ‘co-construction’ of their caregiving identities emerged.^33^ Moreover, providing appropriate, iterative, effective, and collective training was cited as a ‘necessary’ benefit when collaborating with CPCPPs.^31^ Training was reported to support CPCPPs’ involvement in research activities, build their research knowledge in addition to their experiential knowledge, and support their ability to be ‘valuable contributors to the research process’.^31,33^

CPCPPs also shared engagement opportunities regarding their cognitive wellbeing. While CPCPPs expressed the importance of opportunities to gain experience in data collection, they acknowledged it was not always practical or feasible for advanced cancer patients receiving PC to travel to training events.^31^ Similarly, CPCPPs reported considerations regarding limited attention span and restricted physical movement due to advanced stages of cancer, making it important to find tasks that could accommodate their current deteriorated state.^31^

##### Social

Social health is another sub-theme that emerged from our meta-synthesis and can be described as relationship building and understanding, connectedness with others, and community, communication, and support. CPCPPs and experienced researchers reported an appreciation for building and sustaining their ongoing team relationships.^31^ CPCPPs acknowledged how some experienced researchers displayed understanding regarding their unspoken needs and challenging circumstances. For instance, when CPCPPs displayed exhaustion while co-interviewing participants, researchers alleviated CPCPPs from their role; there was ‘an understanding that they could leave the study at any time without giving a reason’^31^ (p. 823).

Other benefits reported by CPCPPs were enhanced self-confidence, feeling a ‘sense of belonging’, and an ‘ability to contribute’ to the research.^32^ CPCPPs expressed that their research engagement enabled them to: (1) achieve personal and collective goals; (2) be part of a supportive community where their experiences were accepted and understood; and (3) be inspired by other CPCPPs as role models.^32^ Similarly, former carers (i.e. CPCPPs) were able to *co-construct* caregiving identities through interactional sequences of shared experiences while co-interviewing current carers with lived cancer and PC experience.^33^

Other forms of social support reported to be important for patient partner engagement included offering alternatives that could fit with their needs and circumstances; for instance: regular check-ins with patient partners to reassess their needs; clearly detailing their partner roles; offering flexible training sessions (individual based and/or as a collective); recording focus groups, so that, those who are unable to attend can listen to them on their own time and still feel connected to the team and involved in the research; and obtain travel insurance to cover the transportation of CPCPPs in researchers’ cars.^31^

There were also opportunities around how to navigate relationships between CPCPPs and experienced researchers, and CPCPPs and participants. CPCPPs perceived there to be power imbalances, which made them feel: (1) undervalued, undermined, used, and marginalized; (2) that their contributions were not perceived as credible; and (3) that they were peripheral to decision-making, core research activities, and priorities.^32^ Furthermore, CPCPPs sensed that the staff had ‘tokenistic attitudes about their involvement’ in the research, and that there could be better job networking opportunities for them.^32^

Due to a lack of understanding and clarity around CPCPPs’ roles, some additional tensions and interactional difficulties in sharing experiences were reported. For example, when CPCPPs spoke about their experiences during interviews, participants would change the topic of conversation and adopt ‘antithetical stances constructing contrary rather than collaborative accounts’^33^ (p. 766). On other occasions, it was reported that the barrier between experienced researchers and CPCPPs became blurred when CPCPPs shared similar experiences and were known to participants.^31^

#### Impact on research

Impacts on the PC research projects due to cancer PC patient partnerships involved *practical* (i.e. recruitment practices; characteristics and retention of engaged patient partners) and *ethical* (i.e. maintaining anonymity) benefits, opportunities, or both.

##### Practical

The sole practical research benefit identified from the collaborative cancer PC patient partnership involved the co-generation of data by both the interviewer (i.e. CPCPPs) and interviewee.^33^ This produced a richer description and understanding of the lived experiences of cancer and PC.

Practical research opportunities that emerged from the meta-synthesis, involved the tensions between balancing the desire to involve CPCPPs at all stages of the research cycle (i.e. from initial design and recruitment, from data collection and analysis, to writing up and dissemination) with the realities advanced CPCPPs face when receiving PC services.^31^ The recruitment and data collection stages with advanced CPCPPs will take longer than is usual or expected; these processes will be interrupted as the CPCPP’s health changes over time, thus impacting the generation, production, and dissemination of meaningful results from the study.^31^ In addition, longer completion times will require additional funding supports and should include the sharing of emerging findings with patient partners over shorter time spans.^31^

##### Ethical

One ethical opportunity that was reported as a research impact involved maintaining participant and CPCPP anonymity. In research, identity anonymity ensures dynamics are not upset and responses inhibited during discussions, such as through focus groups.^33^ However, in the cancer and PC community, it is not uncommon for CPCPPs and research participants to ‘know each other’.^31^

#### Impact on health care system

Due to the original aims of the included studies, we only identified two sub-themes (i.e. ***service improvement*** and ***bureaucratic attitudes and inaction***) focused on the impacts of partnering with cancer PC patients in PC research on the health care system. The one benefit to engaging cancer PC patients as partners in PC research was that CPCPPs perceived their involvement as an ‘opportunity to improve services’,^31–33^ while the one opportunity they reported was the frustration and powerlessness experienced because of ‘bureaucratic staff attitudes’ and ‘professional inaction’.^32^

## Discussion

### Summary of the results

Our systematic review revealed a dearth of research on PE in cancer and PC in general, but especially over the last 7 years, as the most recent studies identified were conducted in 2010 and 2016. While research has been conducted internationally (i.e. the United Kingdom), our synthesis shows that PE has not been applied within other national cancer and PC research contexts. The quality of identified studies, as assessed by CASP^26^ and GRIPP2,^27^ were congruent with all areas of conducting rigorous qualitative research and addressed all the CIHR’s^1^ four pillars of PE; however, there is room for improvement in the reporting of PE to align with the GRIPP2 form checklist.^27^

Our systematic review and meta-synthesis explored and described an array of impacts (i.e. benefits, opportunities) on *patient partners* (i.e. emotional, psychological, cognitive, social), the *research system* (i.e. practical, ethical) and *health care system* (i.e. service improvements, bureaucratic attitudes and inaction), based on established cancer PC research partnerships (Figure 2). All three of the studies included in our systematic review and meta-synthesis engaged CPCPPs as active and equal research team members which coincide with the definitions and foundations of both the UK’s PPI^6^ and CIHR’s SPOR.^34^ Impacts were disproportionately represented in our emergent themes depending on the studies’ original focus and more detailed reporting and transparency of their application of PPI. Still, all themes were derived from our three included studies and are interconnected. For instance, CPCPPs’ engagement in PC research elicits *meaningful contributions* which in turn can *motivate* CPCPPs to fight their cancer and feel a *sense of personal achievement*. Not only were CPCPPs impacted by their engagement, but participants were also impacted by other CPCPPs’ engagement, as were the research and health systems. This is particularly shown through the *inspiration and motivation*, and *relationship and power dynamics* concepts from the *emotional* and *social* health and wellbeing themes.

Some of the impacts to CPCPPs were tied to the research and health system impacts and are consistent with previous health research findings. Like our meta-synthesis, others have reported on how patient partners perceive a sense of personal achievement in paying forward the meaningful contributions they make (i.e. emotional benefits) which, in turn, helps keep them ‘actively distracted’ and able to separate their ‘sense of self’ from the ‘disease’ (i.e. psychological benefits).^35–39^ PE has been found to offer a sense of generativity (*versus* stagnation) in creating an opportunity to build and expand upon research knowledge and skills (i.e. cognitive benefits), and develop a sense of community and belonging (i.e. social benefits).^9,11,40,41^ However, there is evidence that a lack of role clarity can negatively impact the engagement of health research partners and create frustration and emotional distress (i.e. emotional and cognitive opportunities).^7,9^

Noteworthy, there are also unique findings that emerged from our meta-synthesis. For instance, CPCPPs experienced several emotional and physical burdens that inhibited their involvement capacity. CPCPPs reported that sometimes listening to others or recounting their own personal health stories, and experiencing various dismissive and insensitive responses from clinicians and researchers, resulted in distress (i.e. emotional opportunity). CPCPPs expressed that simply possessing a disease diagnosis should not be presumed to equate to their comfort in collecting data from patients with other disease diagnoses (i.e. psychological opportunity). Even though most remained engaged, due to the complex nature of their lived experiences (cancer and PC) and deteriorating health, CPCPPs’ training, involvement capacity, and duration in the research were limited (e.g. cognitive opportunity). Still, co-constructing caregiving identities through interactions and various research innovations (e.g. recording focus groups, paying for travel insurance for transportation), enabled them to remain connected to the team and research process (i.e. social opportunity).

### Systematic review and meta-synthesis study limitations and strengths

Despite our use of rigorous and previously established systematic review methods, there are some study limitations worth considering. First, the differences in language used to define and engage patients as partners in the research studies we reviewed, may have resulted in us missing relevant articles, thereby impacting the comprehensiveness of our synthesis. Relatedly, we may have omitted eligible studies from our synthesis by excluding non-English language studies. Finally, although representative of the current state of the literature, there is a disproportionate representation of relevant studies conducted worldwide; all three of our included studies were conducted in the United Kingdom, which may limit the generalizability of our findings to other geographical contexts.

Aside from these limitations, there are also notable strengths of our work. One strength of this review is that we developed an a priori protocol (ID# CRD42021286744) and submitted it for registration to maintain clarity, transparency, and reproducibility. Second, despite the paucity of published research on the cancer PC patient population, our review findings illustrate the various impacts of engaging them as partners in PC research. Finally, the combination of our convergent and unique findings on CPCPPs suggests feasible methods and recommendations that may enhance future research in this area.

### Recommendations and future directions

Our systematic review and meta-synthesis findings highlight the importance of PE in cancer PC and strengthening research training programs (e.g. training on co-conducting focus groups and interviews) for CPCPPs *and* researchers, to create more accommodating and flexible partnerships throughout the entire research process and to enhance the quality of the data collected. We recommend that future research look at additional, feasible, and effective strategies for engaging CPCPPs in PC research even amid their illness reality. Researchers should ask CPCPPs what they need to be adequately and appropriately supported, prior to their study engagement and subsequently revisit this throughout each stage of their involvement. In addition, we suggest more diverse perspectives (e.g. abilities, ages, ethnicities, gender identities, geographic locations, language groups, racial communities, sexual orientations)^42^ be invited to partner in cancer PC research to ensure equitable and meaningful engagement. Based on observations from our study, Table 9 presents a compiled list of recommendations on the collection and reporting of information for future PE cancer PC research, which should be followed, whenever possible.

**Table 9.** Reporting recommendations.

Recommendation	Who	Details
(1) Demographic information	CPCPPs and participants	Age, PROGRESS Plus^43^ (including place of residence, race, ethnicity, culture, language, occupation, gender/sex, religion, education, socioeconomic status, and social capital)
(2) Years of lived experience of cancer and PC	CPCPPs	
(3) Type of CPCPP	CPCPPs	Type (e.g. patient, family member, caregiver)
(4) Whether CPCPPs are current, former, or both	CPCPPs	
(5) Who CPCPPs cared for, if caregivers and/or family members	CPCPPs	
(6) Whether CPCPPs are currently experiencing cancer and PC		
(7) Specific types and stages of cancer(s) affiliations	CPCPPs	There may be cancer and stage-specific differences of individuals’ ability to partner in PC research
(8) Strategies for identifying and recruiting	CPCPPs and Participants	Including whether CPCPPs joined through an initial research advisory or reference group
(9) Specify what type of CPCPP were engaged at each research stage	CPCPPs	Including what is meant by ‘patient partners were engaged at all stages of the research’

Given the international diversity in the language of PE frameworks, we encourage patient partners and health care researchers to strive for consensus building in nomenclature. We also recommend offering CPCPPs more appropriate, flexible, and adaptable PE frameworks for a more responsive approach to the unique opportunities CPCPPs bring to a PC research project. Manafo *et al.*^2^ state that patient partners are usually engaged in the beginning of the research process. Although Wright *et al.*^31^ and Cotterell *et al.*^32^ engaged CPCPPs at the priority setting stage, all three included studies^31–33^ primarily engaged CPCPPs in the later stages (e.g. data collection) of the PC research projects. This observation calls for an investigation of the specific reasons why CPCPPs were not engaged throughout all stages of the research process.

## Conclusion

Taken together, our study highlights the impacts on CPCPPs and participants involved in PC research and the health care system. Findings from our review highlight the need to consistently apply a PE framework, which might increase the uptake and inclusivity of CPCPPs in future PE cancer PC research. The results of this review can inform the more effective design of cancer PC patient partnerships in PC research and the development of feasible and effective strategies given the cancer and PC context patient partners are coming from.

## Declarations

**Ethics approval and consent to participate:** Not applicable.

**Consent for publication:** Not applicable.

**Availability of data and materials:** The data (i.e. Covidence) from this review can be made available by the authors upon request.

## References

[1] Canadian Institutes of Health Research. Strategy for Patient-Oriented Research – patient engagement framework, https://cihr-irsc.gc.ca/e/48413.html (2019, accessed 14 December 2021).

[2] Manafo E Peterman L Mason-Lai P , et al. Patient engagement in Canada: a scoping review of the ‘how’ and ‘what’ of patient engagement in health research. Health Res Policy Syst 2018; 16: 1–11. 29415734 10.1186/s12961-018-0282-4 PMC5804082

[3] Forbat L Hubbard G Kearney N . Patient and public involvement: models and muddles. J Clin Nurs 2009; 18: 2547–2554. 19207798 10.1111/j.1365-2702.2008.02519.x

[4] Tscherning SC Bekker HL Vedelo TW , et al. How to engage patient partners in health service research: a scoping review protocol. Res Involv Engagem 2021; 7: 1–7. 33902753 10.1186/s40900-021-00268-z PMC8073888

[5] Zibrowski E Carr T McDonald S , et al. A rapid realist review of patient engagement in patient-oriented research and health care system impacts: part one. Res Involv Engagem 2021; 7: 1–13. 34629118 10.1186/s40900-021-00299-6 PMC8504114

[6] National Institute for Health Research (NIHR), INVOLVE, https://www.invo.org.uk (2022, accessed 14 December 2021).

[7] The National Health and Medical Research Council (NHMRC). Consumer and community engagement, https://www.nhmrc.gov.au/about-us/consumer-and-community-engagement (2022, accessed 14 December 2021).

[8] International Collaboration for Participatory Health Research (ICPHR). Promoting the science and enhancing the impact of participatory health research, http://www.icphr.org (2022, accessed 14 December 2021).

[9] Health Research Board (HRB). Public, patient and carer involvement in research, https://www.hrb.ie/funding/funding-schemes/public-patient-and-carer-involvement-in-research/ (2022, accessed 14 December 2021).

[10] Patient-Centered Outcomes Research Institute (PCORI). The value of engagement, https://www.pcori.org/engagement/value-engagement (2022, accessed 14 December 2021).

[11] Israel BA Schulz AJ Parker EA , et al. Community-based participatory research: policy recommendations for promoting a partnership approach in health research. Edu Health 2001; 14: 182–197. 10.1080/13576280110051055 14742017

[12] Poureslami I Pakhale S Lavoie KL , et al. Patients as research partners in chronic obstructive pulmonary disease and asthma research: priorities, challenges, and recommendations from asthma and COPD. Can J Respir Crit Care Sleep Med 2018; 2: 138–146.

[13] Tremblay M-C Bradette-Laplante M Berube D , et al. Engaging indigenous patient partners in patient-oriented research: lessons from a one-year initiative. Res Involv Engagem 2020; 6: 1–11. 32760594 10.1186/s40900-020-00216-3 PMC7376932

[14] Skovlund PC Nielsen BK Thaysen HV , et al. The impact of patient involvement in research: a case study of planning, conduct and dissemination of a clinical, controlled trial. Res Involv Engagem 2020; 6: 1–16. 32699648 10.1186/s40900-020-00214-5 PMC7370448

[15] McCarron TL Clement F Rasiah J , et al. Patients as partners in health research: a scoping review. Health Expect 2021; 24: 1378–1390. 34153165 10.1111/hex.13272 PMC8369093

[16] Brett J Staniszewska S Mockford C , et al. A systematic review of the impact of patient and public involvement on service users, researchers and communities. Patient 2014; 7: 387–395. 25034612 10.1007/s40271-014-0065-0

[17] Ludwig C Graham ID Gifford W , et al. Partnering with frail or seriously ill patients in research: a systematic review. Res Involv Engagem 2020; 6: 1–22. 32944284 10.1186/s40900-020-00225-2 PMC7488581

[18] Chambers E Gardiner C Thompson J , et al. Patient and carer involvement in palliative care research: an integrative qualitative evidence synthesis review. Palliat Med 2019; 33: 969–984. 31250702 10.1177/0269216319858247 PMC6691598

[19] World Health Organization (WHO). Palliative care: fact sheet, https://www.who.int/news-room/fact-sheets/detail/palliative-care (2022, accessed 14 December 2021).

[20] Abel J Kellehear A . Public health palliative care. 1st ed. Oxford: Oxford University Press, 2022.

[21] Page MJ McKenzie JE Bossuyt PM , et al. The PRISMA 2020 statement: an updated guideline for reporting systematic reviews. BMJ 2021; 372: n71. 33782057 10.1136/bmj.n71 PMC8005924

[22] Markle-Reid M Ganaan R Ploeg J , et al. Engagement of older adults with multimorbidity as patient research partners: lessons from a patient-oriented research program. J Multimorbidity Comorbidity 2021; 11: 1–11. 10.1177/2633556521999508 PMC7975523 33796472

[23] Arts and Humanities Research Council (AHRC). ‘Impact summary and pathways to impact frequently asked questions, http://www.ahrc.ac.uk/Funding-Opportunities/Documents/ImpactFAQ.pdf (2010, accessed 14 December 2021).

[24] Cooke A Smith D Booth A . Beyond PICO: the SPIDER tool for qualitative evidence synthesis. Qual Health Res 2012; 2012: 1435–1443. 10.1177/1049732312452938 22829486

[25] Covidence systematic review software, Veritas Health Innovation, Melbourne, Australia, www.covidence.org

[26] Critical Appraisal Skills Program. CASP qualitative checklist, https://casp-uk.b-cdn.net/wp-content/uploads/2018/03/CASP-Qualitative-Checklist-2018_fillable_form.pdf (2018, accessed 14 December 2021).

[27] Staniszewska S Brett J Simera I , et al. GRIPP2 reporting checklists: tools to improve reporting of patient and public involvement in research. BMJ (Clinical research ed.) 2017; 358: j3453. 10.1136/bmj.j3453 PMC5539518 28768629

[28] Hong QN Pluye P Fabregues S , et al. The mixed methods appraisal tool (MMAT) version 2018 for information professionals and researchers. Educ Inf 2018; 34: 285–291.

[29] Sandelowski M Barroso J . Handbook for synthesizing qualitative research. New York: Springer Publishing Company, 2007.

[30] Thomas J Harden A . Methods for the thematic synthesis of qualitative research in systematic reviews. BMC Med Res Methodol 2008; 8: 1471–2288. 10.1186/1471-2288-8-45 PMC2478656 18616818

[31] Wright D Hopkinson JB Corner JL , et al. How to involve cancer patients at the end of life as co-researchers. Palliat Med 2006; 20: 821–827. 17148537 10.1177/0269216306073110

[32] Cotterell P Harlow G Morris C , et al. Service user involvement in cancer care: the impact on service users. Health Expect 2010; 14: 159–169. 21029279 10.1111/j.1369-7625.2010.00627.x PMC5060569

[33] Forbat L Hubbard G . Service user involvement in research may lead to contrary rather than collaborative accounts: findings from a qualitative palliative care study. J Adv Nurs 2015; 72: 759–769. 26689175 10.1111/jan.12865

[34] Canadian Institutes of Health Research. Ethics guidance for developing partnerships with patients and researchers, https://cihr-irsc.gc.ca/e/51910.html (2019, accessed 14 December 2021).

[35] Bhati DK Fitzgerald M Kendall C , et al. Patients’ engagement in primary care research: a case study in a Canadian context. Res Involv Engagem 2020; 6: 1–12. 33292731 10.1186/s40900-020-00238-x PMC7604947

[36] Bird M Ouellete C Whitmore C , et al. Preparing for patient partnership: a scoping review of patient partner engagement and evaluation in research. Health Expect 2020; 23: 523–539. 32157777 10.1111/hex.13040 PMC7321722

[37] Boden C Edmonds AM Porter T , et al. Patient partners’ perspectives of meaningful engagement in synthesis reviews: a patient-oriented rapid review. Health Expect 2021; 24: 1056–1071. 34048618 10.1111/hex.13279 PMC8369105

[38] Bombard Y Baker GR Orlando E , et al. Engaging patients to improve quality of care: a systematic review. Implement Sci 2018; 13: 1–22. 30045735 10.1186/s13012-018-0784-z PMC6060529

[39] Merker VL Hyde JK Herbst A , et al. Evaluating the impacts of patient engagement on health services research teams: lessons from the veteran consulting network. J Gen Intern Med 2022; 37: 33–41. 35349028 10.1007/s11606-021-06987-z PMC8993982

[40] Camden C Shikako-Thomas K Nguyen T , et al. Engaging stakeholders in rehabilitation research: a scoping review of strategies used in partnerships and evaluation of impacts. Disabil Rehabil 2015; 37: 1390–1400. 25243763 10.3109/09638288.2014.963705

[41] Chegini Z Arab-Zozani M Islam SMS , et al. Barriers and facilitators to patient engagement in patient safety from patients and healthcare professionals’ perspectives: a systematic review and meta-synthesis. Nurs Forum 2021; 56: 938–949. 34339525 10.1111/nuf.12635

[42] Manalili K Siad FM Antonio M , et al. Codesigning person-centered quality indicators with diverse communities: a qualitative patient engagement study. Health Expect 2021; 1: 1–15. 10.1111/hex.13388 PMC9615079 34854190

[43] Cochrane. PROGRESS-Plus, https://methods.cochrane.org/equity/projects/evidence-equity/progress-plus (2022, accessed 20 June 2022).

