# Erratum to The impacts of partnering with cancer patients in palliative care research: a systematic review and meta-synthesis

**DOI:** 10.1177/26323524231185693

**Published:** 2023-07-06

Paolucci A, Nielssen I, Tang KL, Sinnarajah A, Simon JE, Santana MJ. The impacts of partnering with cancer patients in palliative care research: a systematic review and meta-synthesis. *Palliative Care and Social Practice*. 2022;16. DOI: 10.1177/26323524221131581

In this article, multiple in-text citations on page 2 and Table 1 have been corrected.

